# Tunable spin and valley dependent magneto-optical absorption in molybdenum disulfide quantum dots

**DOI:** 10.1038/srep41044

**Published:** 2017-01-23

**Authors:** Fanyao Qu, A. C. Dias, Jiyong Fu, L. Villegas-Lelovsky, David L. Azevedo

**Affiliations:** 1 Instituto de Física, Universidade de Brasília, Brasília-DF 70919-970, Brazil; 2 Department of Physics, Qufu Normal University, Qufu, Shandong, 273165, China

## Abstract

Photonic quantum computer, quantum communication, quantum metrology and quantum optical technologies rely on the single-photon source (SPS). However, the SPS with valley-polarization remains elusive and the tunability of magneto-optical transition frequency and emission/absorption intensity is restricted, in spite of being highly in demand for valleytronic applications. Here we report a new class of SPSs based on carriers spatially localized in two-dimensional monolayer transition metal dichalcogenide quantum dots (QDs). We demonstrate that the photons are absorbed (or emitted) in the QDs with distinct energy but definite valley-polarization. The spin-coupled valley-polarization is invariant under either spatial or magnetic quantum quantization. However, the magneto-optical absorption peaks undergo a blue shift as the quantization is enhanced. Moreover, the absorption spectrum pattern changes considerably with a variation of Fermi energy. This together with the controllability of absorption spectrum by spatial and magnetic quantizations, offers the possibility of tuning the magneto-optical properties at will, subject to the *robust* spin-coupled valley polarization.

Carriers in monolayer transition metal dichalcogenides (TMDCs) possess a valley degree of freedom, in addition to charge and spin^1^,^2^. The strong spin-orbit interaction along with spin-valley coupling results in interesting coupled spin-valley pseudospin physics as demonstrated not only experimentally in valley-selective luminescence^3^,^4^, optical generation of valley coherence^3^, valley-resolved magneto-photoluminescence spectroscopy^5^,^6^, spin-coupled valley photocurrent, and in the valley Hall effect^3^,^7^ but also theoretically^8^,^9^,^10^. Since the large separation between K- and *K*′-valley in *k*-space leads to a suppression of intervalley scattering, the valley index renders a promising information carrier in the next generation of photonic quantum computing and optic quantum communications in analogy to the spin in spintronics^11^,^12^. On the other hand, the feasibility of these devices relies on the availability of single-photon source (SPS)^13^, for which extensive relevant studies have been motivated and various single quantum emitters have been demonstrated, e.g., the SPSs based on an atom^14^, molecule^15^, defect or color center and a conventional semiconductor quantum dot (QD)^16^. Very recently, a new class of SPS based on novel 2D materials has been proposed^13^,^17^. However, none of these SPSs generates valley-polarized light which is highly in demand in valleytronic devices. Here we report the band structure and magneto-optic properties of monolayer MoS_2_ circular QDs of the radius *R* subjected to an out-of-plane magnetic field *B* (Fig. 1), which can be created through the top-down nanopatterning of monolayer 2D MoS_2_. We find a gradual transition of the energy spectrum from pure spatial quantization to magnetic quantization, accordingly an evolution from atomic energy levels to Landau levels (LLs). In addition, for the circularly polarized light (CPL), the magneto-optic absorption spectrum of the QDs presents discrete peaks and spin-dependent valley-locked polarization, providing the possibility of SPS when the excited excitons relax. This valley polarization is robust against the variation of QD-geometry and of magnetic field. Furthermore, the excitonic energy of the absorption spectrum is tunable, in contrast to that in the 2D bulk which is uniquely determined by bulk band parameters (e.g., band gap). As for the linearly polarized light (LPL), we find that the absorption spectrum is spin dependent but remains valley unpolarized. To gain deeper insight into magneto-optical properties of MoS_2_ QDs, we also extend our study beyond the independent electron-hole picture and consider the effect of electron-hole interaction (i.e., excitonic effect) on the absorption spectrum.

## Results

### Optical selection rule of TMDC QDs

In order to study the low-energy physics of TMDC monolayers, several research groups have derived an effective **k.p** model in the vicinity of *K (τ* = 1) and *K*′ (*τ* = −1) points^18^,^19^,^20^,^21^. Among them, the two-band model proposed by Xiao *et al*.^18^, which can correctly capture the salient features of the density functional theory calculated band structure and predict/interpret the experimental observations, is widely adopted^4^,^10^,^22^,^23^,^24^. Although the model itself has limitations (as other theories), e.g., it cannot properly describe the spin splitting of conduction band, the distinction of effective mass between conduction and valence bands and the trigonal warping of the spectrum^25^. In the vicinity of the valley *K* or *K*′ that we are interested in and for usual experimental set-ups, these effects play a minor role and can be safely neglected especially in interband optical absorption. For instance, the corrections due to remote bands and different particle-hole masses, respectively appearing as the off-diagonal and diagonal terms in our two-band model, modify the absorption intensity by only around 0.1%^26^. To verify that our two-band **k.p** model (first order)^18^ can well describe the low-energy physics of monolayer MoS_2_, we have performed a comparison of our result, with that calculated by the density functional theory (DFT)^27^ and that obtained by the **k.p** models of second and third orders^19^,^21^,^25^,^28^, see Supplementary material (SM), Sec. I. The simplicity but reliability of the two-band effective model motivates us to utilize it in our calculation about magneto-optical absorption spectrum. We give a more detailed description about the two-band model later on in Sec. Method. Here we only focus our attention on the optical selection rule.

We assume that the TMDC QDs are exposed to light fields with the energy *ħω* and wave vector **k**_*l*_ which is orthogonal to the plane of QDs and much smaller than 1/*a*. Up to the 1st order approximation, the light-matter interaction Hamiltonian is described by,


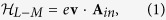


with *e* > 0 the electron charge, the light field 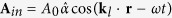, and 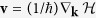 being propagation velocity. Note that in the vicinity of *K* and *K*′ valleys, i.e., **k** · **r** ≪ 1, we assume 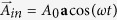. Here *A*_0_ and 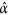 stand for the amplitude and orientation of the polarization field, respectively. For an electron being excited by an incident photon from its initial state |*i*〉 to a final state | *f* 〉, the transition probability is given by the Fermi’s golden rule as, 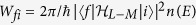, where *n(E*) is the density of states available for the final state. The absorption intensity *I* can be evaluated by,


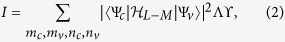


where Ψ_*c*_ (Ψ_*v*_) is the conduction (valence) band wave function, Λ = *γ*/*π*{[*ω* − (*E*_*c*_(*m*_*c*_, *n*_*c*_) − *E*_*v*_(*m*_*v*_, *n*_*v*_))]^2^ + *γ*^2^}, and ϒ = *f*_*c*_ − *f*_*v*_, with *f*_*i*_ Fermi-Dirac distribution function, *n*_*i*_ the principle quantum number, *m*_*i*_ the quantum number associating with orbital angular momentum, *i* = *c* and *v* referring to conduction and valence band, respectively, *m*_*c*_ = *m* and *m*_*v*_ = *m*′ (see Method), *γ* a parameter determined by the Lorentzian distribution. Note that at *T* = 0 K one has ϒ = *θ(E*_*F*_ − *E*_*c*_(*m*_*c*_, *n*_*c*_)) − *θ(E*_*F*_ − *E*_*v*_(*m*_*v*_, *n*_*v*_)), with *θ(x*) the heaviside function.

For the CPL, 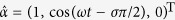, with *σ* = ±1 denoting the corresponding positive and negative helicities and T stands for the transpose of a matrix, then the perturbed Hamiltoninan becomes,


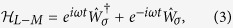


where 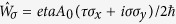, with *t* the effective hopping integral and *a* the lattice constant. Accordingly, the optical transition matrix elements in the QDs are computed by,


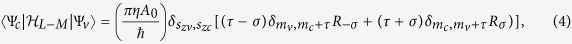


where *s*_*zv*_ (*s*_*zc*_) denotes the valence (conduction) band spin state, 
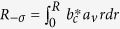
 and 
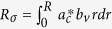
 with *a*_*c/v*_ and *b*_*c/v*_ the radial components of the conduction/valence band spinor (see Method). The selection rule for optical transitions in TMDC QDs are defined by *m*_*v*_ − *m*_*c*_ = ±*τ* and *s*_*zv*_ = *s*_*zc*_, i.e., the angular momentum of the initial and final states differs by ±1, but the spin of these two states is the same. The magnitude of transition rates is determined by the integral *R*_*−σ*_ and *R*_*σ*_ for the transitions taking place in the valley *τ* = −*σ* and *σ*, respectively. Since |*a*_*c*_(*r*)| > |*b*_*c*_(*r*)| and |*a*_*v*_(*r*)| < |*b*_*v*_(*r*)|, the integral *R*_−*σ*_ is much smaller than the *R*_*σ*_. As a consequence, the absorption in the valley *τ* = *σ* is stronger than that in the *τ* = −*σ*, which leads to a valley selected absorption. In a special case in which one component of the wave function spinor is equal to zero such as *n*_*ll*_ = 0 LL, only photons with the helicity *σ* = *τ* is absorbed, one obtains a dichroism *η* = 1, see SM, Secs. II and III.

For the LPL, there is no valley polarization in the absorption spectrum (see SM, Sec. IV), in contrast to the case of CPL.

### Optical absorption of TMDC QDs excited by circularly polarized light at zero field

#### Zero-field energy levels of TDMC QDs

**Figure 2:**
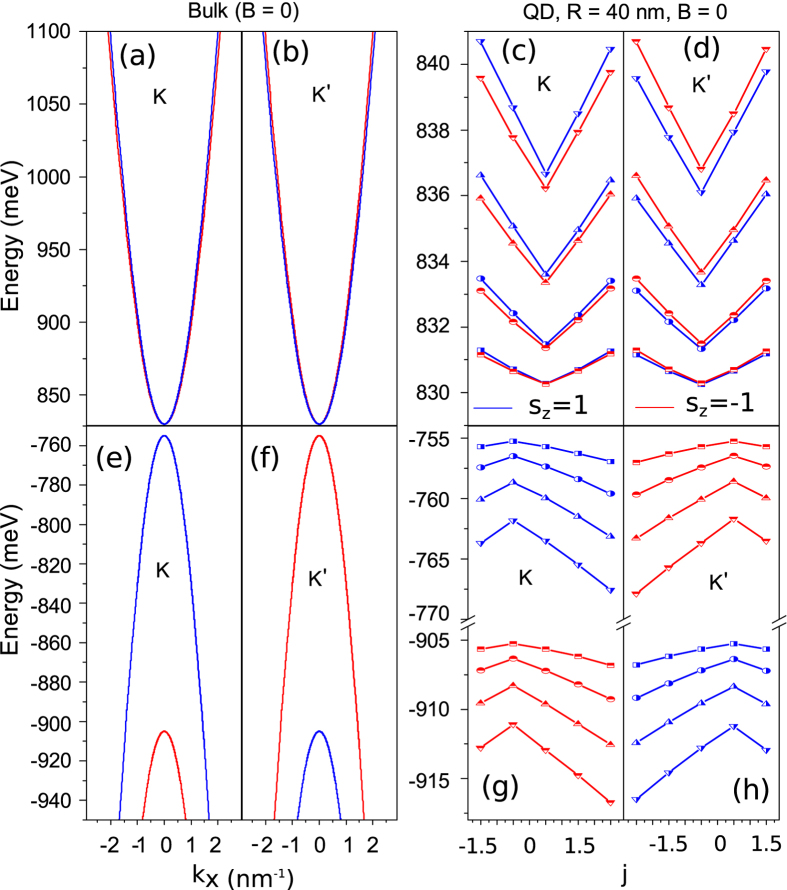
Zero-field conduction-band energy spectrum near the *K* (**a**) and *K*′ (**b**) points of the 2D bulk MoS_2_ and the lowest four conduction band energy levels of a 40-nm QD in the valleys *K* (**c**) and *K*′ (**d**), for both the spin up (blue curves) and spin down (red curves) states. (**e**–**h**) The corresponding energy spectra for the valence band.

Figure 2(a) and (b) show zero-field conduction band energy spectrum of 2D monolayer MoS_2_ for both the spin-up and spin-down states in the *K* and *K*′ valleys, respectively. At either the *K* or *K*′ point (i.e., *k* = 0), the energy level has a four-fold degeneracy. Away from them, the spin degeneracy of the electronic states in a given valley is lifted due to the coupling between conduction and valence bands. Nevertheless, the energy of the states with an opposite value of *k*_*x*_ for a given valley and spin is the same. In addition, the energy spectrum in the *K*-valley presents time-reversal symmetry (TRS) with that in *K*′ valley. In Fig. 2(c) and (d), we show the lowest four conduction band energy levels of a QD with *R* = 40 nm as functions of the angular momentum *j* (see Method), in the *K* and *K*′ valleys, respectively. In contrast to that of the 2D monolayer MoS_2_, the splitting of the spin-up and spin-down states in the same valley emerges for every value of *j*. The splitting is more pronounced in a higher energy subband and for a larger value of *j*. Since there is no direct spin-orbit term for the conduction band in the *K* (or *K*′) valley in our model as we mentioned above, we attribute the spin splitting to the dot confinement potential. Physically, the spin splitting arises from the fact that the Hamiltonian does not commute with the spin-orbit term. In addition, since the dot confinement potential couples with the orbital pseudospin, the *effective* time reversal symmetry is broken within a single valley even in the absence of a magnetic field, similar to graphene quantum dots/rings^29^. In contrast with 2D bulk, for a given valley and spin, the energy of the states with an opposite value of *j* is different, i.e., *E*^*τ*^(*j*) ≠ *E*^*τ*^(−*j*). Nevertheless, the equality *E*^*τ*^(*m*) = *E*^*−τ*^(−*m*) involving two distinct valleys still holds due to the real TRS at zero magnetic field, cf. Fig. 2(c) and (d), as expected.

Figure 2(e–h) show the valence band structure of the 2D monolayer bulk MoS_2_ and the QD in the *K* and *K*′ valleys. A breaking of particle-hole symmetry is observed. It manifests itself in a large spin-orbit splitting. This is attributed to the fact that the Hamiltonian of the 2D bulk MoS_2_ as well as the QD do not commute with the *effective* inversion symmetry operator defined in a single valley given by *P*_*e*_ = *I*_*τ*_ ⊗ *σ*_*x*_, with *I*_*τ*_ the identity matrix. However, for a given spin, the main feature of quantum confinement effect on the energy spectrum is similar to that of the conduction band.

#### Tunable valley- polarized optical absorption of TMDC QDs

**Figure 3:**
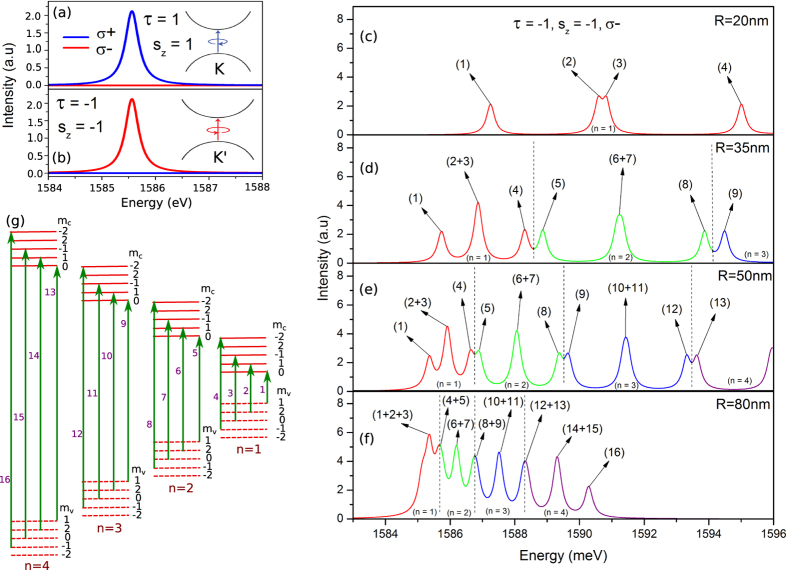
Zero-field optical absorption spectrum associated with interband transitions between conduction- and valence-band ground states of a 70-nm dot for the spin-up state in the *K*-valley **(a**) and for the spin-down state in the *K*′-valley (**b**), pumped by both clockwise (*σ*^+^, blue curve) and anticlockwise (*σ*^−^, red curve) circularly polarized light fields. (**c**–**f**) Absorption intensity for the spin-down state in the *K*′-valley under the excitation of *σ*^−^, for QDs of *R* = 20, 35, 50, and 80 nm, respectively. (**g**) Schematic diagram of the involved interband transitions in (**c**–**f**) tagged by the numbers. The color of the curves is used to highlight the principle quantum number (*n*) of transition involved states.

In the 2D bulk MoS_2_, the bottom of the conduction band at the two valleys is characterized by the orbital angular momentum *m* = 0. In contrast, at the top of the valence band, the orbitals with *m* = 2 in the *K*-valley, while *m* = −2 in the *K*′-valley. The valley dependent angular momentum in the valence band allows one to address different valleys by controlling the photon angular momentum, i.e., by the helicity of the CPL. Such valley-specific circular dichroism of interband transitions in the 2D bulk has been confirmed^7^,^11^,^18^. The question is whether its counterpart QD also possesses this exotic optical property. Figure 3(a) and (b) depict zero-field band edge optical absorption spectrum of a 70-nm dot pumped by the CPL field. Interestingly, one notices that (i) the polarization of the absorption spectrum is locked with the valley degree of freedom, manifested by the intensity of absorption spectrum with *σ* = *τ* being about 10^6^ times stronger than that with *σ* = −*τ*, and (ii) the spectrum is spin-polarized. Thus, the QDs indeed inherit the valley and spin dependent optical selection rule from their counterpart of 2D bulk MoS_2_. In spite of the distinction in the spin- and valley- polarization of absorption spectra in the distinct valleys, their patterns are the same required by the TRS.

In Fig. 3(c–f) we show the zero-field optical absorption spectrum as a function of excitation energy for several values of dot-radius within *R* = 20–80 nm. The involved transitions in Fig. 3(c–f) lagged by the numbers have been schematically illustrated in Fig. 3(g). Alike conventional semiconductor QDs, several peaks stemmed from discrete excitations of the MoS_2_ QD are observed. As the dot-size decreases, the peaks of absorption spectrum undergo a blue shift. In other words, a reduction of the dot-size pushes the electron excitations to take place between higher energy states, as a result of the enhancement of the confinement on carriers induced by a shrink of the dot. Therefore, the spin-coupled valley selective absorption with a tunable transition frequency can be achieved in QDs by varying dot geometry, in contrast to the 2D bulk where a fixed transition frequency is uniquely determined by the bulk band structure. In addition to the blue shift of the transition frequency, the absorption intensity can also be controlled by dot geometry due to size dependence of the peak interval. In fact, an increasing of dot size results in a reduction of the energy separation among confined states. Thus as the dot size increases, the absorption peaks gets closer and closer, cf. Fig. 3(c–f). Eventually, several individual absorption peaks merge together to yield a single composite-peak with an enhanced intensity. For instance, for the 20-nm dot [Fig. 3(c)], the lowest energy peak is generated by only one transition, labeled by (1) [see also Fig. 3(g)]. However, for the dot with *R* = 80 nm, we observe a highly enhanced absorption intensity labeled by (1 + 2 + 3), which in the 20-nm dot refers to three separated peaks with weak absorption intensities indicated by (1), (2), (3), respectively.

### Magneto-optical absorption of TMDC QDs excited by circularly polarized light at nonzero field

#### Energy levels of the TDMC QDs subjected to an applied magnetic field

**Figure 4:**
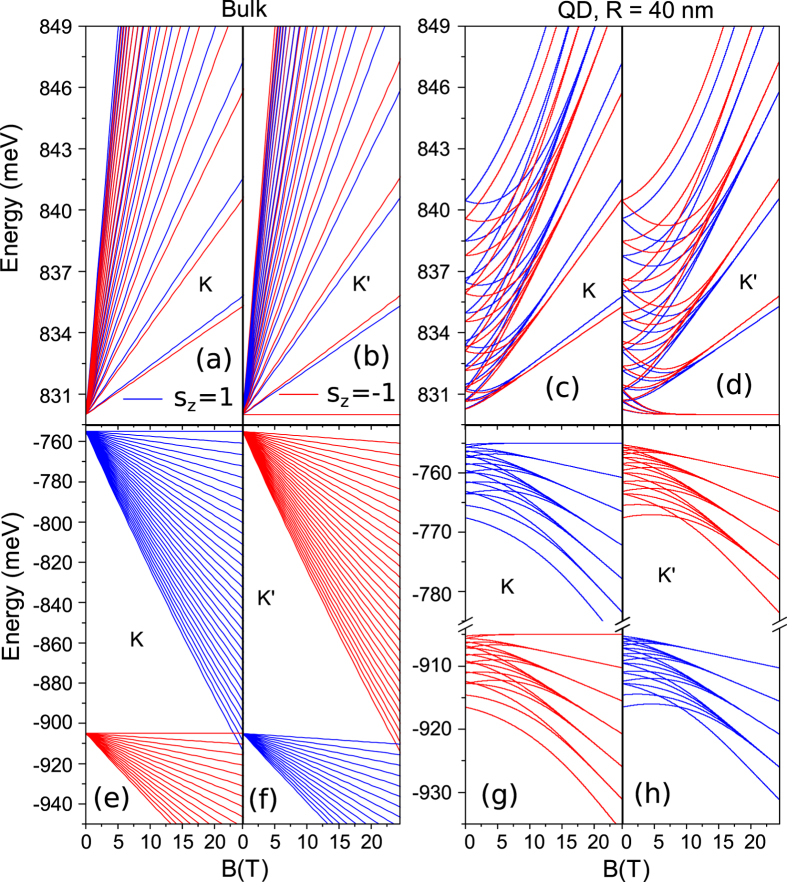
Conduction-band energy spectrum near the valleys *K* (**a**) and *K*′ (**b**) in the 2D bulk MoS_2_ and energy levels of the lowest four conduction bands of a 40-nm QD in the valleys *K* (**c**) and *K*′ (**d**), for both the spin up (blue curves) and spin down (red curves) states, as a function of magnetic field. (**e**–**h**) The corresponding dependence of the valence-band energy spectrum on the magnetic field.

In order to gain insight into energy levels of QDs subjected to a magnetic field perpendicular to the 2D plane, as a reference Fig. 4(a) and (b) show the fan diagram of the conduction band of the 2D bulk MoS_2_ as a function of magnetic field, in the *K* and *K*′ valleys, respectively. Because of the large effective mass at the band edges the LLs of the bulk TMDC resemble that of conventional semiconductors. On the other hand, unlike the conventional semiconductor, there is *n*_*ll*_ = 0 LL lying at the bottom of the conduction band in the valley *K*′, as shown in Fig. 4(b), lifting valley degeneracy of the ground state. The corresponding analogues to Fig. 4(a) and (b) but for a QD of *R* = 40 nm are shown in Fig. 4(c) and (d). In contrast to the fan diagram of the 2D bulk where the energies of the LL increases monotonically with magnetic field, the energy levels of the QDs undergo a gradual transition from pure spatial quantization to dominating magnetic quantization. More specifically, in the regime of low magnetic field, the valley degenerated atomic structure is pronounced, where the energy of the dot is distinct from that of the 2D bulk. With increasing *B*, the energy levels from different orbitals of the dot start to merge together, leading to an enhanced degeneracy. A further increasing of *B* leads to more energy branches being assembled and eventually the formation of the LLs. Consequently, the magnetic fan will be born, where the energy in the bulk TMDs and QDs becomes identical because the cyclotron energy predominates over the atomic energy. In addition, if one compares energy levels in the *K*-valley with that in the *K*′-valley, a surprising behavior will be observed. Two LLs with the same index number *n*_*ll*_ but lying in different valley are composed of different coalesced branches. Let us pick up *n*_*ll*_ = 1 LL as an example, it is comprised of the orbitals of *j* = −3/2, −1/2, 0 and *s*_*z*_ = 1/2 in the *K*-valley while *j* = −3/2, −1/2, 1/2, 3/2, and *s*_*z*_ = 1/2 orbitals in the *K*′-valley.

Figure 4(e–h) display valence-band energy spectrum of the 2D bulk MoS_2_ and the QD of *R* = 40 nm in the presence of a perpendicular magnetic field. Notice that the general feature of magnetic field effects on the energy levels of the dot is similar to that in the conduction band such as there is *n*_*ll*_ = 0 LL lying at the top of the valence band in the *K*-valley, as shown in Fig. 4(e). Interestingly, the separation between the *n*_*ll*_ = 0 LL in the conduction band and the one in the valence band can be used as an effective tool to measure the optical band gap of the TMDCs. On the other hand, unlike the conduction band, in a given valley, the strong spin-orbit coupling splits the LLs into well separated spin-up and spin-down groups. In the high-field regime, e.g., *B* ~ 25 T for the 2D bulk in the parameter range considered here, there are crossings between the high-energy LLs of one spin state and the low-energy LLs of the opposite spin state. The crossing of two group LLs results in enhanced degeneracies^30^. Similarly, for the QD, if we account for more LLs in Fig. 4(g) and (h), there is also a clear LL crossing, inherited from the 2D bulk. In order to visibly display the transition of our dots from atomic levels to LLs, here we did not show enough number of LLs to feature the LL crossing.

#### Tunable spin-coupled valley-polarized magneto-optical absorption in TMDC QDs

**Figure 5:**
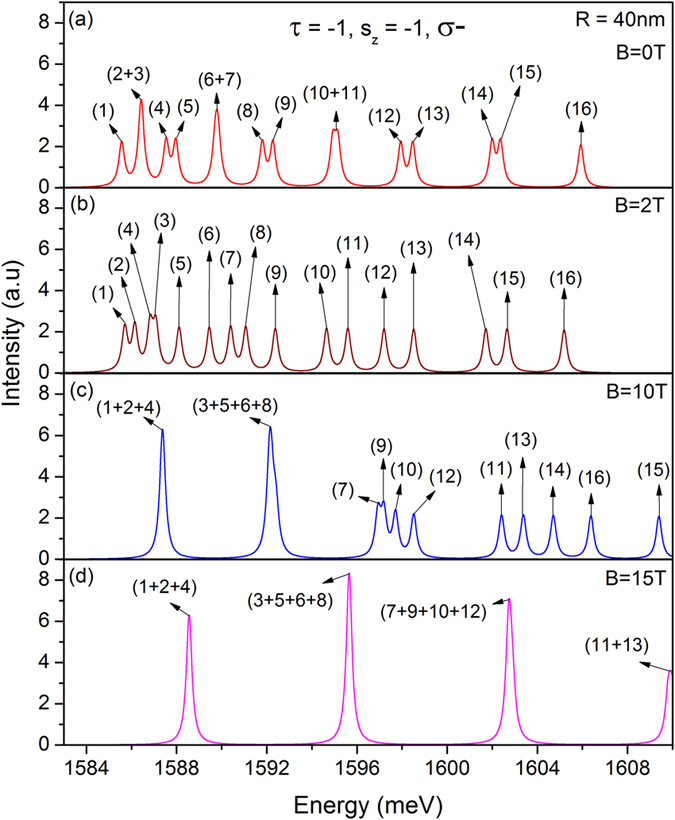
Absorption spectrum of a QD with *R* = 40 nm for the spin-down state in the *K*′-valley under the excitation of circularly polarized light *σ*^−^, at the magnetic field *B* = 0 (**a**), 2 T (**b**), 10 T (**c**), 15 T (**d**), respectively. The corresponding enumerated optical transitions are schematically shown in Fig. 3(g).

With the knowledge of energy levels of the QDs under the magnetic field, we are ready to study their magneto-optical properties. Figure 5 shows the magneto-optical absorption spectra of a QD with *R* = 40 nm for the spin down state in the *K*′ valley (i.e., *τ* = −1, *s*_*z*_ = −1) under the excitation of *σ*_−_ light field, for several values of magnetic field ranging from 0–15 T. A few interesting features are observed. Firstly, the magneto-optical absorption is also spin- and valley-dependent, as it does in optical excitation spectrum at zero field. In particular the lowest excitation-energy absorption peak related to the interband transition involving *n*_*ll*_ = 0 LL is totally valley polarized with dichroism equal to 1. Thus the polarization of magneto-optic absorption locks with the valley. Secondly, for a fixed value of dot-size, increasing (decreasing) the strength of the magnetic field results in a blue (red) shift in the absorption spectrum. This arises from the fact that the magnetic field induces an *effective* confinement characterized by the magnetic length 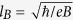, which is more pronounced for a stronger magnetic field. In addition, the magnetic field induced magnetic quantization *forces* the quantum dot to absorb photons with higher energies. The stronger the magnetic field, the greater the capacity of the QDs to absorb the photons of higher energy. Thirdly, in the high magnetic-field regime the absorption intensity can be highly enhanced by increasing the strength of magnetic field due to increased degeneracy of the LLs.

### Absorption spectrum of TMDC QDs excited by linearly polarized light

In contrast to the case of CPL excitation, where the valley degree of freedom can be selectively accessed by optical helicity, we observe that the LPL field generates comparable optical transitions in the *K* and *K*′ valleys. Accordingly, the total absorption intensity in magnitude under LPL is around twice of that under CPL. For a detailed comparison of absorption spectrum between CPL and LPL fields, see SM, Sec. V.

### Externally controlled optical absorption of TMDC QDs

In addition to the control of the optical absorption by dot geometry as we discussed above, here we report another alternative method, i.e., by tuning the Fermi level (*E*_*F*_) of QDs. We find that the characteristic of the valley and spin selective optical absorption is independent of the Fermi level. However, in either *K* or *K*′ valley, the number of absorption peaks decreases when the Fermi level is lifted. For a detailed discussion on the effect of Fermi level on optical properties, see SM, Sec. VI.

### Excitonic effect

The approach we have adopted so far to view optical absorption processes does not account for the electron-hole interaction. Although the independent electron-hole picture has been used to successfully investigate optical and magneto-optical properties of monolayer TMDCs^8^,^31^, in order to gain deeper insight into absorption spectra, we go beyond this approximation and consider the effect of electron-hole interaction (i.e., excitonic effect)^32^,^33^. More specifically, we consider the two-dimensional many-body Hamiltonian, which takes into account both direct and exchange contributions of the electron-hole Coulomb interaction, and obtain the exciton binding energy *E*_*b*_ ~ 550 meV in our dots, close to the value (500 meV) experimentally reported^34^. Our result shows a considerable exciton absorption peak below the band-edge absorption. And, the excitonic absorption peak shifts monotonically to higher absorption energy as the dot size is increased. On the other hand, above the band gap, the absorption spectrum resembles what we discussed above from the independent electron-hole picture, consistent with the description about excitonic absorptions by Yu and Cardona^35^. We shall emphasize that the valley selective polarization we discussed above based on the independent electron-hole picture is also valid even accounting for the excitonic effect. For more details about the excitonic effect on the absorption spectrum, see SM, Sec. VII.

## Discussion

Optical and magneto optical properties of the MoS_2_ QDs strongly depend on the polarization of incident light fields. On the one hand, we observe that QDs pumped by the CPL inherit the spin and valley selected circular polarization from the 2D bulk; On the other hand, for the LPL, the optical absorption is only spin dependent but valley unpolarized. Moreover, in addition to the control of magneto-optical properties by dot geometry, the absorption spectrum can also be tuned by a variation of the Fermi level, which is achievable by changing the dopping condition and/or a gate voltage. It is worth emphasizing that the emission spectrum of the QDs is closely related to its counterpart, i.e., absorption spectrum. Either of them can be used to demonstrate the optical properties of QDs. Here in this work we only focus on the absorption spectrum. As far as for the excitonic effect, an additional peak associated with the exciton absorption located around 550 meV below the band-edge obsorption emerges. The presence of the exciton peak only slightly affects the band-edge absorption governed by the electronic structure of QDs, while it does not change the valley selectivity. Furthermore, since the exciton absorption peak is far away from the band-edge absorption, one can in principle study them separately, which is what we have done in this work. As a final remark, here we consider relatively large dots of tens of nm. However, for small dots especially when the dot size is comparable to the lattice constant, the intervally effect may cause the valley depolariztion. More work is needed to investigate this interesting possibility as well as other sources leading to the loss of valley polarization, e.g., valley exchange interaction^36^.

## Methods

We first focus on the single-particle picture. Let us start from recalling the modified effective Hamiltonian for monolayer MoS_2_, which applies generally to other group TMDCs having the same crystal structure. The top and bottom *S* layers and the middle Mo layer are parallel triangular lattices. In the two inequivalent *K* and *K*′ valleys at the corners of the hexagonal Brillouin zone, the conduction band (CB) arises from 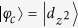 orbital, while the valence band (VB) is approximately from hybridization of 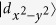 and |*d*_*xy*_〉 orbitals, i.e., 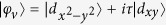. Then in the vicinity of the *K* and *K*′ valleys, wave functions can be constructed by using |*φ*_*c*_〉 and |*φ*_*v*_〉 as basis functions, i.e., the monolayer MoS_2_ can be described by an effective two-band Hamiltonian. With this effective Hamiltonian at hand, it is straightforward to write down the low-energy effective Hamiltonian for our QDs,


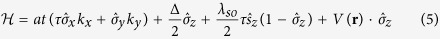


where 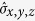 and 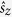 are the Pauli matrices, acting on the orbital and spin spaces, respectively. 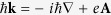 is the kinetic momentum, with **A** the vector potential describing a magnetic field, Δ the energy gap, and *λ*_*so*_ the spin-orbit coupling constant. *V*(**r**) is the confinement potential creating our dot, which has circular shape. We assume *V*(**r**) = 0 for |**r**| ≤ *R* and *V*(**r**) → ∞ for |**r**| > *R*. The confinement potential can be produced either by the lateral heterojunctions between two different TMDCs with different gaps within a single uniform crystalline monolayer or by lateral confinement potentials on an extended monolayer, e.g. by patterned electrodes^19^. The following typical parameters for a monolayer MoS_2_, such as *a* = 0.3193 nm, *t* = 1100 meV, Δ = 1660 meV, and *λ*_*so*_ = 75 meV are used in this work.

Owing to the rotational symmetry of *V*(**r**), 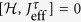, here *j* is determined by the total angular momentum operator 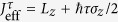 and *L*_*z*_ is the orbital angular momentum along the *z* direction. The eigenfunction of Eq. 5 admits the *Ansatz*, 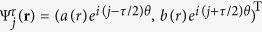, where (*r, θ*) are the polar coordinates, *j* the total angular momentum, and the superscript T refers to the transpose of a matrix. The eigenvalue problem 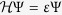 is solved under the symmetric gauge, i.e., 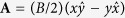, for an out-of-plane magnetic field 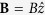, here *ε* denotes eigenvalue. After some algebraic calculations, one derives the following analytic expression for the eigenfunction,


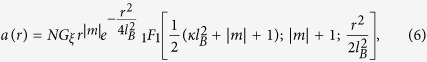


and


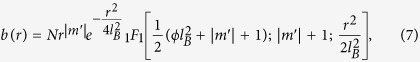


where we have defined 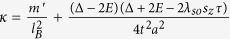, 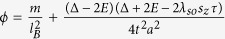, the effective total angular momentum *j* = *m* + *τ*/2 depending on the valleys with *m* the orbital angular momentum, *m*′ = *j* + *τ*/2, 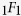 the confluent hypergeometric function of the first kind, *N* the normalization constant, and *G*_*ξ*_ a complex constant depending on the total angular momentum *j*, energy *ε*, valley index *τ* and spin *s*_*z*_. To solve this eigenvalue problem, we adopt the infinite mass boundary condition^29^ *b(R*)/*a(R*) = *iτ*, which is determined by imposing that the radial component of the probability current vanishes at *r* = *R*. After some algebraic calculations, we obtain the following eigenvalue equation,


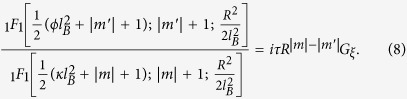


For the eigenvalue problem in the special case of *B* = 0, we obtain, 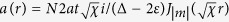 and 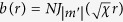, with *J* the Bessel function of the first kind. The eigenvalue equation is, 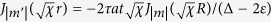. Here we have defined *χ* = (2*ε* − Δ)(Δ + 2*ε* − 2*λ*_*so*_*s*_*z*_*τ*)/4*t*^2^*a*^2^ and *N* the normalization constant.

To account for the excitonic effect, we add the electron-hole Coulomb interaction, *V*^*e*−*h*^(**r**_*e*_ − **r**_*h*_) = (1/4*πε*_*r*_*ε*_0_)(*e*^2^/|**r**_*e*_ − **r**_*h*_|), into the single-partilce Hamilontian for electron and hole 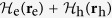 (Equation 5). Here *ε*_0_ is the permittivity, *ε*_*r*_ is the dielectric constant, and **r**_*e*_ and **r**_*h*_ respectively stand for the position of electron and hole. For a detailed implementaion of many-body effects, see SM, Sec. VII.

## Additional Information

**How to cite this article**: Qu, F. *et al*. Tunable spin and valley dependent magneto-optical absorption in molybdenum disulfide quantum dots. *Sci. Rep.* **7**, 41044; doi: 10.1038/srep41044 (2017).

**Publisher's note:** Springer Nature remains neutral with regard to jurisdictional claims in published maps and institutional affiliations.

## Supplementary Material

Supplementary Information

## Figures and Tables

**Figure 1:**
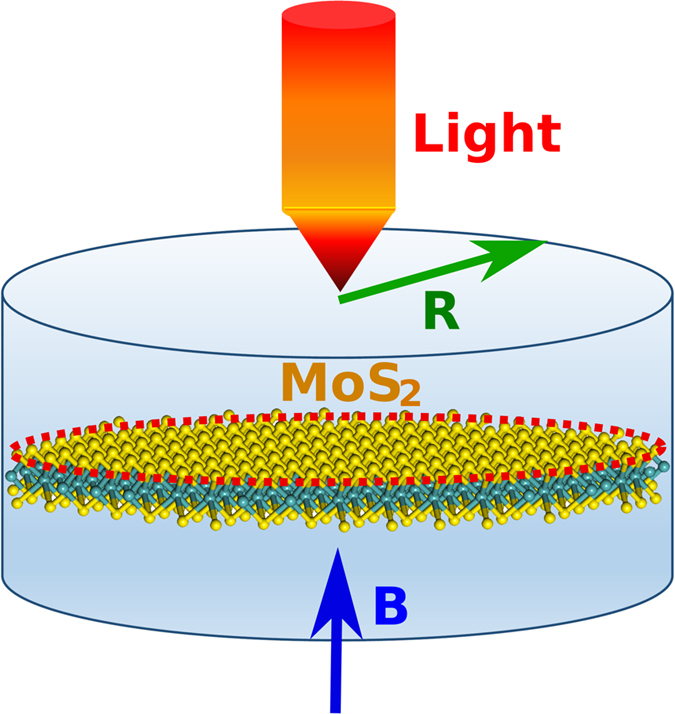
Schematic of a monolayer MoS_2_ circular quantum dot with radius *R* indicated by a red circle, excited by a light field. A magnetic field *B* is applied perpendicularly to the MoS_2_ sheet.
