# Supplementary material for: Tunable spin and valley dependent magneto-optical absorption in molybdenum disulfide quantum dots
Source: Sci Rep. 2017 Jan 23;7:41044. doi: 10.1038/srep41044 (PMC5253649; doi:10.1038/srep41044)
Supplement: Supplementary Information [file srep41044-s1.pdf]

Supplementary Material

Tunable spin and valley dependent magneto-optical absorption in molybdenum disulfide quantum dots

Fanyao Qu,[1,*] A. C. Dias,[1] Jiyong Fu,[1,2,†] L. Villegas-Lelovsky,[1] and David L. Azevedo[1]

[1]*Instituto de Física, Universidade de Brasília, Brasília-DF 70919-970, Brazil*

[2]*Department of Physics, Qufu Normal University, Qufu, Shandong, 273165, China*

[*] fanyao@unb.br

[†] yongjf@mail.qfnu.edu.cn

I. BAND DISPERSION: A COMPARISON AMONG DIFFERENT METHODS

To verify that our two-band **k**.**p** model (first order)[1] can well describe the low-energy physics of monolayer MoS_2, we have performed a comparison of our result, with that calculated by the density functional theory (DFT)[2] and that obtained by the **k**.**p** models of second and third orders[3–6], as shown in Fig. S1. We emphasize that our DFT calculation is implemented by using the quantum espresso package[2]. The electronic wave functions are expanded in a plane-wave basis set with the energy cutoff of 1360 eV. The core of atoms is replaced by fully relativistic pesudopotentials with an additional information for spin-orbit calculations. The valence states used for Mo and S pseudoatoms are $4d^5 5s^1$ and $3s^2 3p^4$, respectively. The exchange-correlation effects are included through the generalized gradient approximation (GGA) proposed by Perdew et al.[7]. For the Brillouin-zone sampling, we use the 16×16×1 Monkhorst-Pack mesh. The energy tolerance for geometry optimization convergence is set as 1.0×10^{-8} a.u. We find that, near the K (or K') valley our result agrees very well with that produced by the DFT and other **k**.**p** models. This provides a strong support for us to perform our calculation using the two-band model.

II. LANDAU LEVELS OF 2D BULK TMDCS.

For a comparison of the energy spectrum in QDs with that in the 2D bulk with infinite geometry, we derive the analytic solution of the bulk energy spectrum,

$$E_{\pm,n_{ll}} = \frac{\lambda_{so}\tau s_z}{2} \pm \sqrt{\frac{(\Delta - \lambda_{so}\tau s_z)^2}{4} + t^2 a^2 \omega_c^2 n_{ll}}, \tag{S1}$$

where t denotes the effective hopping integral, a is the lattice constant, Δ is the energy gap, λ_{so} is the spin-orbit coupling constant, s_z stands for the electron spin, τ refers to the valley index, n_{ll} is the Landau level (LL) index, and $\omega_c = \sqrt{2}/l_B$, with $l_B = \sqrt{\hbar/eB}$ the magnetic length. Note that for the n_{ll}=0 LL, $E_{+,0} = \Delta/2$ and $E_{-,0} = -\Delta/2 + \lambda_{so}\tau s_z$. The calculated eigenstates indicate that the zeroth LL $E_{+,0}$ is located at the bottom of conduction band in the K'-valley, while the zeroth LL $E_{-,0}$ lies at the top of valence band in the K-valley. Both of them are flat bands with respect to the magnetic filed B.

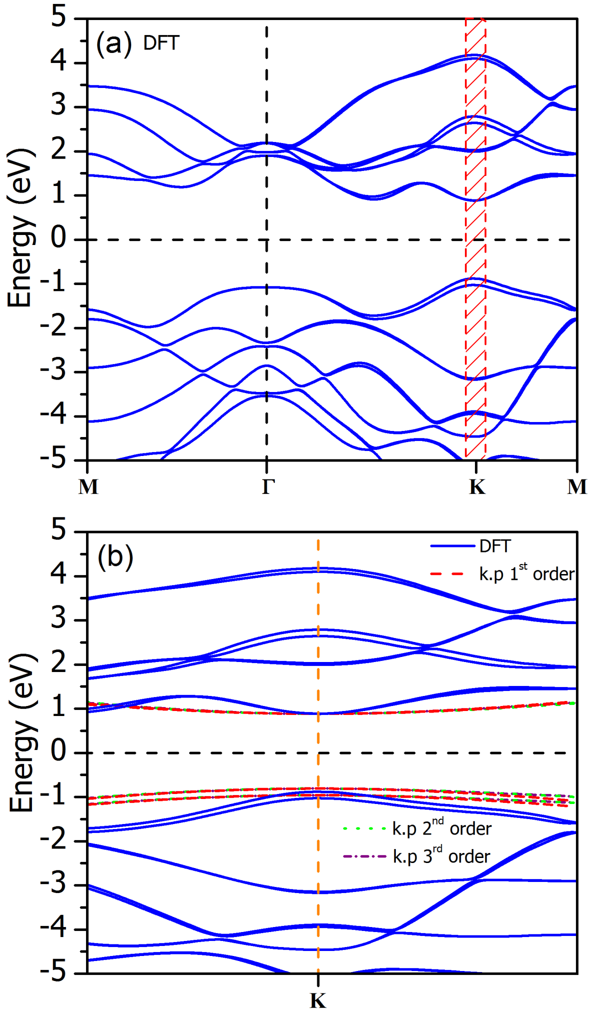

FIG. S1. (Color online) (a) Quasiparticle band structure of monolayer MoS_2 calculated by the density functional theory (DFT)[2]. For a detailed implementation of our DFT calculation, see Sec. I. (b) A blowup of the rectangular red area in (a). The blue (solid), red (dashed), green (dotted) and purple (dot-dashed) curves correspond to the results obtained by the DFT, and **k.p** theory of the first order[1], second order[3–6], and third order[3–6], respectively. Notice that around the K-point, all methods give almost identical results.

III. VALLEY POLARIZED OPTICAL ABSORPTION SPECTRUM OF 2D BULK TMDCS

To understand optical properties of QDs at zero field, let us recall that of 2D bulk materials. In this case, the eigenvalues for the conduction (+) and valence (−) bands are given by

$$E_{\pm} = \frac{\lambda_{so}\tau s_z}{2} \pm \sqrt{\frac{(\Delta - \lambda_{so}\tau s_z)^2}{4} + t^2a^2k^2}, \tag{S2}$$

and the correspondent eigenstates read

$$|c,\mathbf{k},\tau,s_z\rangle = |s_z\rangle \otimes \begin{pmatrix} \cos(\frac{\vartheta_n}{2}) \\ \tau\sin(\frac{\vartheta_n}{2})e^{i\tau\phi_{\mathbf{k}}} \end{pmatrix}, \qquad |v,\mathbf{k},\tau,s_z\rangle = |s_z\rangle \otimes \begin{pmatrix} -\tau\sin(\frac{\vartheta_n}{2})e^{-i\tau\phi_{\mathbf{k}}} \\ \cos(\frac{\vartheta_n}{2}) \end{pmatrix}, \tag{S3}$$

where c and v denote the conduction and valence bands, respectively, and $\tan(\phi_{\mathbf{k}}) = k_y/k_x$, $\cos\vartheta_n = (\Delta + (-1)^n\lambda_{so})/2\sqrt{(\Delta + (-1)^n\lambda_{so})^2 + 4t^2a^2k^2}$. Then the optical transition matrix elements between the states with $\tau = 1$ and $s_z = 1$ can be evaluated by $P^{cv}_{\pm} = \langle c,\mathbf{k}|p_x \pm ip_y|v,\mathbf{k}\rangle$. It is straightforward to obtain $P^{cv}_{+} = 2m_0at(\sin\phi_{\mathbf{k}} + \cos\phi_{\mathbf{k}}\cos\vartheta_n)/\hbar$ and $P^{cv}_{-} = 0$. Accordingly, the dichroism $\eta = (|P^{cv}_{+}|^2 - |P^{cv}_{-}|^2) = 1$.

IV. TMDC QDS EXCITED BY A LINEARLY POLARIZED LIGHT.

For a linearly polarized light (LPL), its field orientation is defined as, $\hat{\alpha} = (\alpha_x, \alpha_y, 0)^{\mathrm{T}}$ with T denots the transpose of a matrix. The form of the Hamiltonian describing the light-matter interaction is the same as that in Eq. (3) in the main manuscript for a circularly polarized light (CPL), but with $\hat{W} = \eta A_0(\alpha_x\tau\sigma_x + \alpha_y\sigma_y)/\hbar$. Then we obtain the transition matrix element,

$$\langle\Psi_c|\mathcal{H}_{L-M}|\Psi_v\rangle = \left(\frac{2\pi\eta A_0}{\hbar}\right)\delta_{s_{zv},s_{zc}}[(\tau_z\alpha_x + i\alpha_y)\delta_{m_v,m_c+\tau_z}R_{-\sigma} + (\tau_z\alpha_x - i\alpha_y)\delta_{m_c,m_v+\tau_z}R_{\sigma}]. \tag{S4}$$

Equation S4 clearly shows that the selection rule for LPL excited optical transitions in the TMDC QDs is also defined by $m_v - m_c = \pm\tau$ and $s_{zv} = s_{zc}$, the same as that in the CPL case, see the main manuscript. In addition, similar to the CPL excitation, the magnitude of a transition rates is also determined by the integral $R_{-\sigma}$ and R_{σ} for the transition in the $\tau = -\sigma$ and $\tau = \sigma$ valley, respectively. However, there is no valley polarization in the absorption spectrum, as opposed to the CPL case.

V. ABSORPTION SPECTRA OF TMDC QDS EXCITED BY CIRCULARLY AND LINEARLY POLARIZED LIGHT

Now let us turn to the absorption spectrum of the QDs under a linearly polarized light. In order to gain insight into dependence of optical absorption of the QDs on incident light-polarization, in Fig. S2 we show the absorption spectra for both the spin up and spin down states under the σ^+ CPL, σ^- CPL and LPL fields, in the K and K' valleys of the 70-nm dot at zero magnetic field. A total contribution to the absorption spectrum from both valleys, which may refer to the real experimental data, is also shown. Obviously, the LPL field generates comparable optical excitations in the two valleys, i.e., there are observable optical transitions in either K or K' valley, in contrast to the CPL field, where the valley degree of freedom can be selectively accessed by optical helicity (i.e., σ^+ and σ^-). Therefore, the total absorption intensity under LPL is around twice of that under CPL.

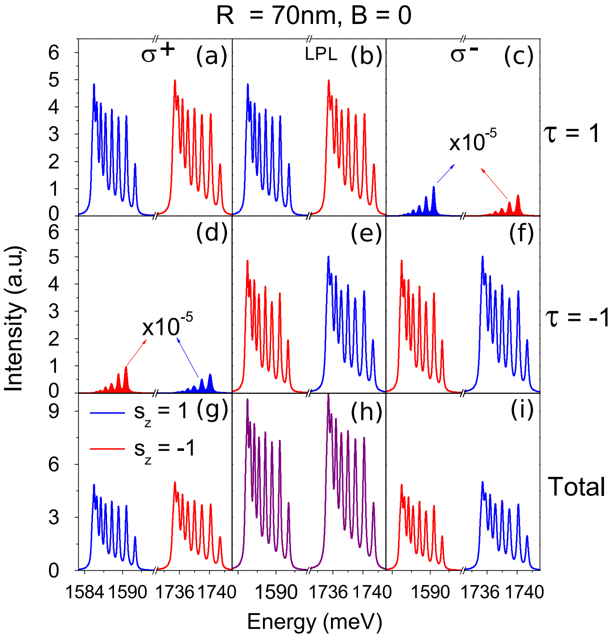

FIG. S2. (Color online) Zero-field optical absorption spectrum of the 70-nm dot in the K valley for both the spin-up (blue curves) and spin-down (red curves) states, under clockwise circularly polarized light σ^+ (a), linearly polarized light (b), and anti-clockwise circularly polarized light σ^- (c). The corresponding analogues in the K' valley and the total contribution from both valleys are shown in (d)-(f) and (g)-(i), respectively. The magnetic field is chosen as zero.

Let us move to the effect of magnetic field. In Fig. S3, we show the magneto-optical absorption spectra of the 70-nm dot under both CPL and LPL fields at $B = 8$ T. Since the magnetic field induces an extra magnetic confinement in addition to the dot confinement

potential, we observe that the absorption peaks with larger separation than those at $B = 0$, cf. Figs. S2 and S3, Moreover, the formation of degenerated LLs in a strong magnetic field leads to enhanced absorption intensity which depends on the degeneracy of LLs. This is manifestation of collective contribution of the branches of which LLs is comprised.

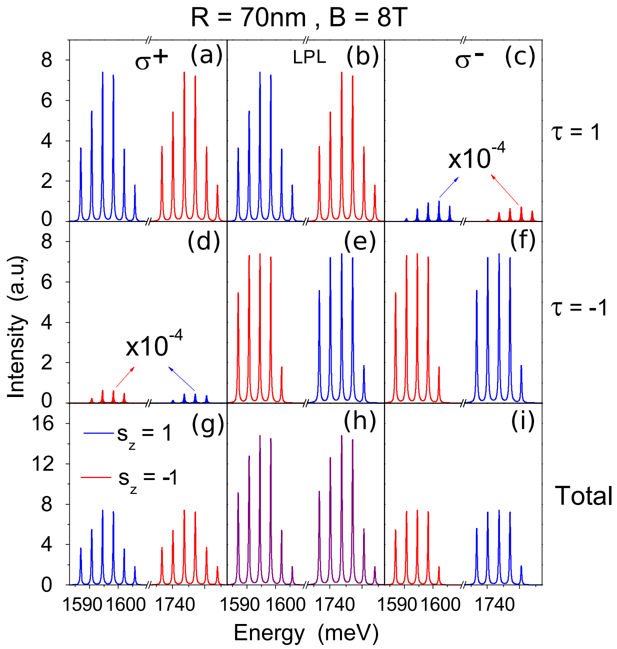

FIG. S3. (Color online) Magneto-optical absorption spectrum of the 70-nm dot in the K valley for both the spin-up (blue curves) and spin-down (red curves) states, under clockwise CPL σ^+ (a), LPL (b), and anti-clockwise CPL σ^- (c). The corresponding analogues in the K' valley and the total contribution from both valleys are shown in (d)-(f) and (g)-(i), respectively. The magnetic field is chosen as $B = 8$ T.

VI. EXTERNALLY CONTROLLED OPTICAL ABSORPTION OF TMDC QDS.

In our discussions above, we have focused our attention on QD-geometry tunable optical and magneto-optical properties. Below, we report another alternative method, i.e., by tuning the Fermi level (E_F) of QDs, to control the absorption spectrum. Figures S4(a)-S4(c) show the absorption spectrum of the 70-nm dot for the (K, spin up) state, for three values of Fermi levels, with $E_F = 0, 831, 832$ meV, respectively. The corresponding results in the K' valley for the spin down state are shown in Figs. S4(d)-S4(f). It is found that the valley and spin selective optical selection rule is independent of the Fermi level, as expected. However, in either K or K' valley, the number of absorption peaks decreases when the Fermi level is lifted. It is attributed to an increase in the number of filled final states which prohibit the

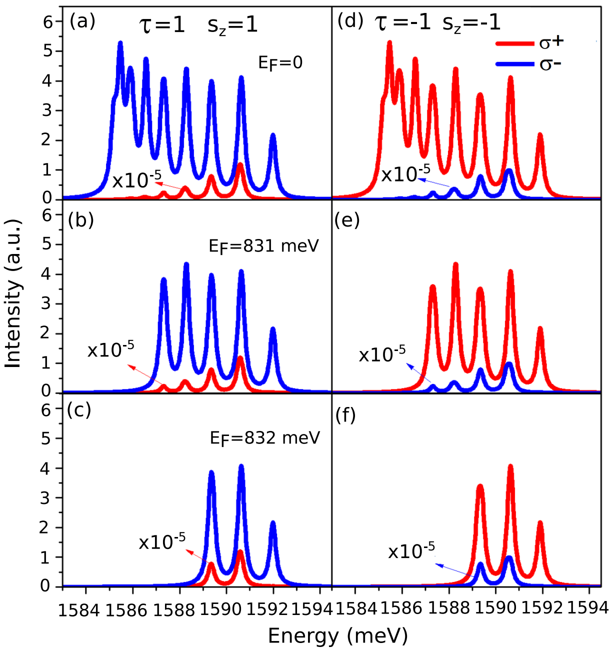

FIG. S4. (Color online) Optical absorption spectrum (zero field) accounting for higher energy transitions, for the (K, spin up) state (a)-(c) and (K', spin down) state (d)-(f), in a QD of $R = 70$ nm under circularly polarized light fields of both σ^+ and σ^-. Three values of Fermi energy are considered, with $E_F = 0, 831, 832$ meV, respectively.

optical transitions involving them. The Fermi level dependence of the optical absorption allows for a control of the absorption spectrum, through the dopping and/or a gate voltage.

VII. EXCITONIC EFFECT

The optical and magneto-optical absorptions we discussed so far is based on the assumption of the independent electron-hole picture. In reality, there is a strong Coulomb interaction between the electron and hole in MoS_2. The full treatment of the electron-hole interaction depends upon many-body theory, which is beyond the scope of this paper. However, the excitonic effects in MoS_2 QDs can be addressed by an exact diagonalization. The Hamiltonian used to describe the exciton is, $H(\mathbf{r}_\mathrm{e}, \mathbf{r}_\mathrm{h}) = \mathcal{H}_\mathrm{e}(\mathbf{r}_\mathrm{e}) + \mathcal{H}_\mathrm{h}(\mathbf{r}_\mathrm{h}) + V^{\mathrm{e-h}}(\mathbf{r}_\mathrm{e} - \mathbf{r}_\mathrm{h})$, where $\mathcal{H}_{\mathrm{e(h)}}$ is the single electron (hole) Hamiltonian in QDs (see Eq. 5 in main text) and $V^{\mathrm{e-h}}$ is the electron-hole Coulomb interaction described by $V^{\mathrm{e-h}}(\mathbf{r}_\mathrm{e} - \mathbf{r}_\mathrm{h}) = (1/4\pi\epsilon_r\epsilon_0)(e^2/|\mathbf{r}_\mathrm{e} - \mathbf{r}_\mathrm{h})|)$. Here ϵ_0 is the permittivity, ϵ_r is the dielectric constant, and $\mathbf{r}_e$ and $\mathbf{r}_h$ respectively stand for the position of electron and hole.

An exciton can be understood as a coherent combination of electron-hole pairs. Thus, the wave funciton of an exciton can be constructed based on a direct product of single-particle wave functions for the electron and hole (independent electron-hole picture). Due to the strong excitonic effect, here we use the modified single-particle wave function by a hydrogen-like s-wave state depending on the exciton Bohr radius, i.e., $\chi_j(\mathbf{r}_{\rm e,h}) = N\exp(-r_{\rm e,h}/r_b)\Psi_j(\mathbf{r}_{\rm e,h})$, where N is the normalization constant, Ψ_j is the wave fucntion of the Hamiltonian $\mathcal{H}_{\rm e,h}$, and r_b is the exciton bohr radius, which has the value of $r_b \sim 1$ nm in MoS_2.[8] Then, the exciton wave function Ψ_{exc} can be straightforwardly written as,

$$\Psi^{\nu}_{exc}(\mathbf{r}_{\rm e}, \mathbf{r}_{\rm h}) = \sum_{i,j} C^{\nu}_{i,j} \;\; \chi_i(\mathbf{r}_{\rm e}) \; \chi_j(\mathbf{r}_{\rm h}), \tag{S5}$$

with the superscript ν referring to the ν-th exciton state.

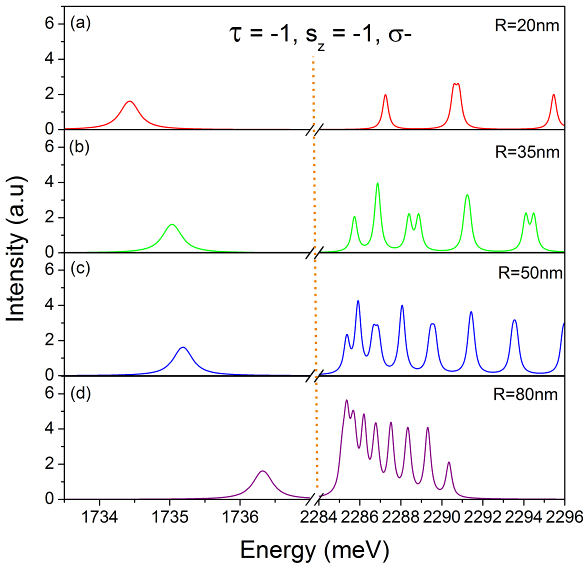

FIG. S5. (Color online) Optical absorption spectrum (zero field) accounting for the excitonic effect, for the (K', spin down) state, in QDs of $R = 20$ nm (a), 35 nm (b), 50 nm (c), and 80 nm (d) under the circularly polarized light field of σ^-. The Fermi energy is chosen as 0. The vertical dashed line marks two distinct regions of the absorption spectrum: left-hand side for excitonic absorption and right-hand side for single-particle like absorption. To be consistent with the experimental report of excitonic absorption energy, a right shift of photon energy of 700 meV is made.

Within the Hilbert space made of states of electron-hole pairs $\{\chi_i(\mathbf{r}_{\rm e})\chi_j(\mathbf{r}_{\rm h})\}$, the matrix

element of Coulomb integral reads,

$$V_{prsq}^{\text{e}-\text{h}} = -\frac{e^2}{4\pi\epsilon_0\epsilon_r}\int\int \frac{\chi_p^*(\mathbf{r}_\text{e})\chi_r^*(\mathbf{r}_\text{h})\chi_s(\mathbf{r}_\text{h})\chi_q(\mathbf{r}_\text{e})}{|\mathbf{r}_\text{e}-\mathbf{r}_\text{h}|}d\mathbf{r}_\text{e}d\mathbf{r}_\text{h}, \tag{S6}$$

which involves the wave function χ_i of the electron and hole in the absence of Coulomb interaction, with $j = p, r, s, q$ indicating the state index. We should emphasize that Eq. S6 contains both the direct interaction between the electron and hole, i.e., Eq. S6 at $p = q$ and $r = s$,

$$V_{prrp}^{\text{e}-\text{h,dir}} = -\frac{e^2}{4\pi\epsilon_0\epsilon_r}\int\int \frac{|\chi_p(\mathbf{r}_\text{e})|^2|\chi_r(\mathbf{r}_\text{h})|^2}{|\mathbf{r}_\text{e}-\mathbf{r}_\text{h}|}d\mathbf{r}_\text{e}d\mathbf{r}_\text{h}, \tag{S7}$$

and the exchange interaction, i.e., Eq. S6 at $s = p$ and $r = q$,

$$V_{prpr}^{\text{e}-\text{h,ex}} = -\frac{e^2}{4\pi\epsilon_0\epsilon_r}\int\int \frac{\chi_p^*(\mathbf{r}_\text{e})\chi_r^*(\mathbf{r}_\text{h})\chi_p(\mathbf{r}_\text{h})\chi_r(\mathbf{r}_\text{e})}{|\mathbf{r}_\text{e}-\mathbf{r}_\text{h}|}d\mathbf{r}_\text{e}d\mathbf{r}_\text{h}. \tag{S8}$$

To determine the Coulomb potential described in Eq. S6, in our calculation we expand $1/|\mathbf{r}_\text{e}-\mathbf{r}_\text{h}|$ in terms of half-integer Legendre function of the second kind $Q_{m-1/2}$, i.e.,

$$\frac{1}{|\mathbf{r}_\text{e}-\mathbf{r}_\text{h}|} = \frac{1}{\pi\sqrt{r_\text{e}r_\text{h}}}\sum_{m=0}^{\infty}\epsilon_m\cos[m(\theta_\text{e}-\theta_\text{h})]\ Q_{m-1/2}(\xi), \tag{S9}$$

which is widely used in many-body calculations.[9] Here $\theta_{\text{e(h)}}$ is the polar angle of the position vector $\mathbf{r}_{\text{e(h)}}$ in the 2D plane of MoS_2, $\xi = (r_\text{e}^2 + r_h^2)/2r_\text{e}r_\text{h}$, $\epsilon_m = 1$ for $m = 0$ and $\epsilon_m = 2$ for $m \neq 0$.

The exciton energy and the corresponding wavefunction can be obtained by an exact diagonalization of the many-particle Hamiltonian $H(\mathbf{r}_\text{e}, \mathbf{r}_\text{h})$. With the exciton state at hand (Eq. S5), we are ready to the determine the excitonic absorption involving a transition from the ground state $|0\rangle$ to exciton state $|f\rangle = |\Psi_{exc}\rangle$,

$$A(\omega) = \sum_f |\langle 0|\mathcal{P}|f\rangle|\ \delta\{\hbar\omega - E_{exc}^{\nu}\}, \tag{S10}$$

where $\mathcal{P} = \sum_{i,j}\delta_{sz,sz'}\langle\chi_i|\mathcal{H}_{L-M}|\chi_j\rangle\ a_{i,sz}\ h_{j,sz'}$ is the polarization operator, with $a_{i,sz}$ and $h_{j,sz'}$ the electron and hole annihilation operators, respectively,[10] E_{exc}^{ν} the exciton energy, and

$\hbar\omega$ the photon energy. By inserting Eq. S5 into Eq. S10, one has,

$$A(\omega) = \sum_{\nu} \left(\delta_{sz,sz'} \sum_{i,j} C^{\nu}_{i,j} \langle \chi_i | \mathcal{H}_{L-M} | \chi_j \rangle \right) \delta\{\hbar\omega - E^{\nu}_{exc}\}. \tag{S11}$$

In our numerical calculations, we consider five modified single-particle basis functions of distinct angular momentum ranging from -2.5 to 1.5, see Method section in the main text. From Fig. S5, we find that our calculated excitonic absorption spectrum of MoS_2 QDs shows an exciton absorption peak located at around 550 meV (i.e., exciton binding energy) below the band-edge absorption. And, the excitonic absorption peak shifts monotonically to higher absorption energy as the dot size is increased. Above the band gap, however, the spectrum is similar to what we considered earlier in the band-to-band transitions using the independent electron-hole model. Since the exciton absorption peak is far away from the band-edge absorption, one can in principle study them separately. And, the Coulomb interaction between electron-hole pair does not change the valley selectivity and our general conclusion. Finally, it is worth to remarking that our model calculation allows one to shift the exciton peak to a higher energy side in order to fit experimental data by varying the band gap parameter.

---

[1] Xiao, D. *et al.* Coupled spin and valley physics in monolayers of MoS_2 and other group-VI dichalcogenides. *Phys. Rev. Lett.* **108**, 196802 (2012).

[2] Giannozzi, P. *et al.* Quantum espresso: a modular and open-source software project for quantum simulations of materials. *J. Phys.: Condens. Matter* **21**, 395502 (2009).

[3] Liu, G. B. *et al.* Three-band tight-binding model for monolayers of group-vib transition metal dichalcogenides. *Phys. Rev. B* **88**, 085433 (2013).

[4] Kormányos, A. *et al.* Spin-orbit coupling, quantum dots, and qubits in monolayer transition metal dichalcogenides. *Phys. Rev. X* **4**, 011034 (2014).

[5] Kormányos, A. *et al.* Monolayer MoS_2: Trigonal warping, the Γ valley, and spin-orbit coupling effects. *Phys. Rev. B* **88**, 045416 (2013).

[6] Kormányos, A. *et al.* **k.p** theory for two-dimensional transition metal dichalcogenide semiconductors. *2D Mater.* **2**, 022001 (2015).

[7] Perdew, J. P. *et al.* Generalized gradient approximation made simple. *Phys. Rev. Lett.* **77**, 3865 (1996).

[8] Mak, K. F. *et al.* Tightly bound trions in monolayer MoS_2. *Nature Materials* **12**, 207 (2013).

[9] Cohl, H. S. *et al.* Useful alternative to the multipole expansion of 1/r potentials. *Phys. Rev. A* **64**, 052509 (2001).

[10] Güçlü, A. D., Potasz, P. & Hawrylak, P. Excitonic absorption in gate-controlled graphene quantum dots. *Phys. Rev. B* **82**, 155445 (2010).
